# Janus kinase inhibitor ruxolitinib in combination with nilotinib and prednisone in patients with myelofibrosis (RuNiC study): A phase Ib, multicenter study

**DOI:** 10.1002/jha2.685

**Published:** 2023-04-16

**Authors:** Rosa Ayala, Rafael Alonso Fernández, Valentín García‐Gutiérrez, Alberto Alvarez‐Larrán, Santiago Osorio, Jose M. Sánchez‐Pina, Gonzalo Carreño‐Tarragona, Noemi Álvarez, María Teresa Gómez‐Casares, Antonia Duran, Julian Gorrochategi, Juan Carlos Hernández‐Boluda, Joaquín Martínez‐López

**Affiliations:** ^1^ Haematological Malignancies Clinical Research Unit Hospital Universitario 12 de Octubre, Universidad Complutense, CNIO, CIBERONC Madrid Spain; ^2^ Hematology Department Hospital Universitario 12 de Octubre Madrid Spain; ^3^ Hematology Department Hospital Universitario Ramón y Cajal Madrid Spain; ^4^ Hematology Department Hospital ClíNic Barcelona Spain; ^5^ Hematology Department Hospital General U Gregorio Marañón Madrid Spain; ^6^ Department of Translational Hematology Research Institute Hospital 12 de Octubre (i+12) Madrid Spain; ^7^ Hematology Department Hospital Universitario de Gran Canaria Dr. Negrin Las Palmas de Gran Canaria Spain; ^8^ Hematology Department Hospital Universitario Son Espases Palma de Mallorca Spain; ^9^ Vivia Biotech S.L. Tres Cantos Madrid Spain; ^10^ Hospital Clínico Universitario‐INCLIVA Valencia Spain

**Keywords:** combination therapy, myelofibrosis, nilotinib, prednisone, ruxolitinib

## Abstract

This phase Ib, non‐randomized, open‐label study evaluates the safety and tolerability of ruxolitinib in combination with nilotinib and prednisone in patients with naïve or ruxolitinib‐resistant myelofibrosis (MF). A total of 15 patients with primary or secondary MF received the study treatment; 13 patients had received prior ruxolitinib treatment (86.7%). Eight patients completed seven cycles (53.3%) and six patients completed twelve cycles of treatment (40%). All the patients experienced at least one adverse event (AE) during the study (the most common AEs were hyperglycemia, asthenia, and thrombocytopenia), and 14 patients registered at least one treatment‐related AE (the most common treatment‐related AEs were hyperglycemia (22.2%; three grade 3 cases). Five treatment‐related serious AEs (SAEs) were reported in two patients (13.3%). No deaths were registered throughout the study. No dose‐limiting toxicity was observed. Four out of fifteen (27%) patients experienced a 100% spleen size reduction at Cycle 7, and two additional patients achieved a >50% spleen size reduction, representing an overall response rate of 40% at Cycle 7. In conclusion, the tolerability of this combination was acceptable, and hyperglycemia was the most frequent treatment‐related AE. Ruxolitinib in combination with nilotinib and prednisone showed relevant clinical activity in patients with MF. This trial was registered with EudraCT Number 2016‐005214‐21.

## 1 INTRODUCTION

Myelofibrosis (MF) is characterized by the clonal expansion of myeloid cells due to the acquisition of driver mutations in the *JAK2, CALR*, or *MPL* genes, and/or other mutations such as those in *ASXL1, SRSF2*, and *U2AF1* [1, 2].

Ruxolitinib is a reversible and selective inhibitor of Janus kinase‐1 (JAK1) and JAK2/signal transducer and activator of transcription (STAT) signaling that has been approved in the European Union for the treatment of disease‐related splenomegaly or symptoms in adult patients with de novo MF, MF arising from previous polycythemia vera, or essential thrombocythemia (MF) [3, 4, 5, 6, 7, 8, 9, 10, 11]. Ruxolitinib achieves spleen reduction and symptom control in a significant proportion of patients, leading to improved quality of life and possible survival benefits. However, the median response duration for ruxolitinib of 3 years, its lack of disease‐modifying effect, and treatment discontinuation due to adverse events (AEs) highlight the need to incorporate new treatment options for patients with MF [5, 6, 7, 8, 9, 10, 11].

A recent in vitro study investigated the administration of ruxolitinib in combination with nilotinib and prednisone to treat patients with MF [12] and showed that ruxolitinib had a synergistic effect with nilotinib and prednisone in MF patient samples and cell lines, partly mediated through significant inhibition of the STAT5 and protein kinase B (AKT/PKB) pathways. Moreover, the combination of ruxolitinib and nilotinib has proven efficacy in chronic myeloid leukemia, and nilotinib has shown antifibrotic effects in the liver and lung in animal models [13, 14, 15, 16].

The present phase Ib clinical trial was a two‐arm study designed to obtain and assess the maximum tolerated dose (MTD) and/or recommended phase III dose (RP3D) of ruxolitinib in combination with nilotinib and prednisone in two MF separate populations: patients naïve to JAK‐inhibitor treatment and patients not responding to or relapsed after JAK‐inhibitor treatment. The study was also designed to provide preliminary efficacy and safety data for ruxolitinib when administered in combination with nilotinib and prednisone in MF patients.

## 2 METHODS

### 2.1 Patient population

This study consisted of two cohorts: patients naïve to JAK‐inhibitor treatment and patients who did not respond or relapsed after JAK‐inhibitor treatment. Patients not responding to or who relapsed after 12 weeks of JAK‐inhibitor treatment were defined according to the international Working group for Myelofibrosis Research and Treatment criteria (IWG‐MRT)(ref Tefferi A, Blood 2013;122): patients who did not achieved a splenic response measured by palpation (<50% reduction) or symptoms measured by modified MF‐SAF (<50% reduction) or had > 50% of spleen size increase from best response or >50% increase in symptoms assessment from best response. A detailed explanation of the key inclusion and exclusion criteria is included in the Supplementary Material.

### 2.2 Study design

**FIGURE 1:**
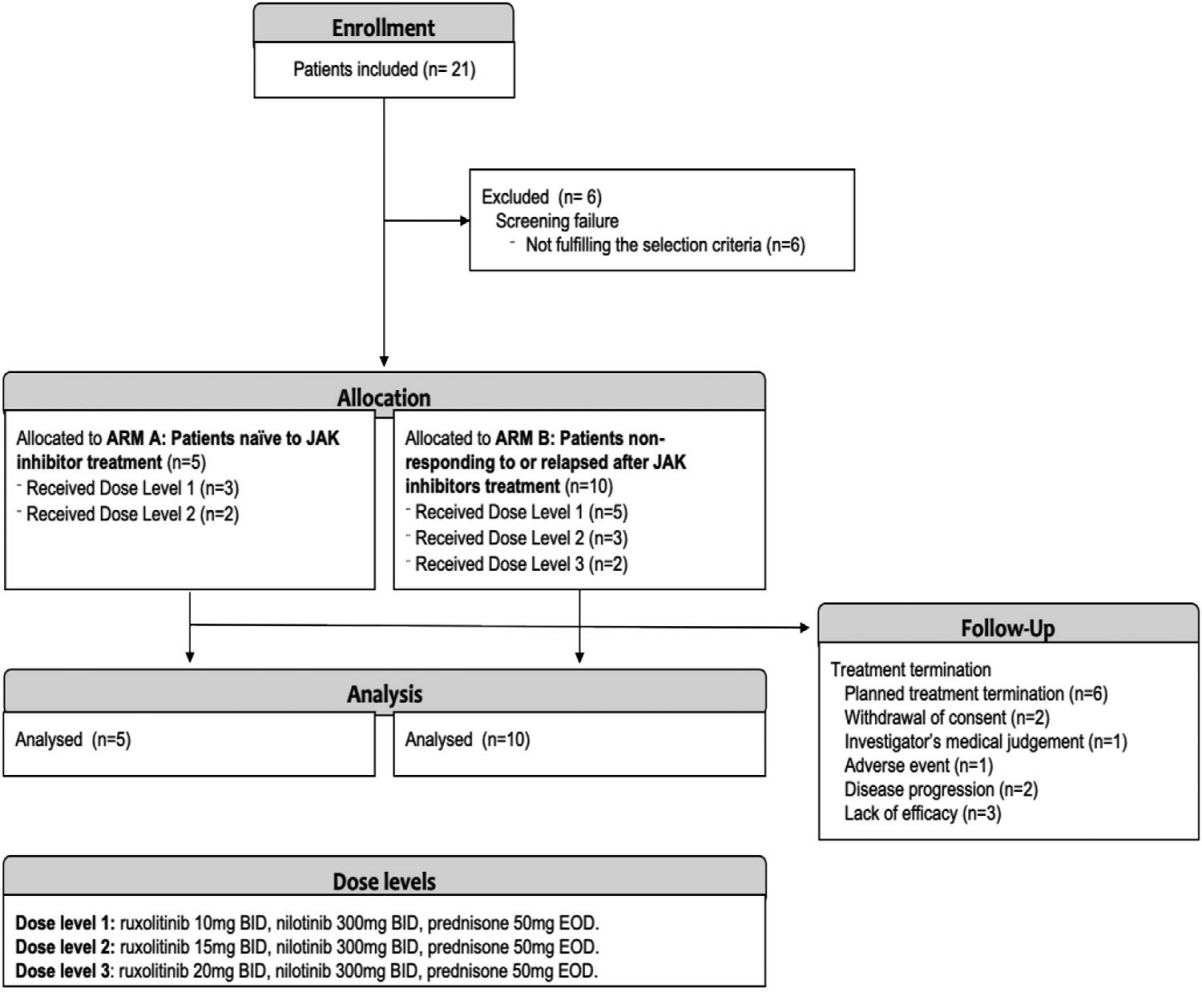
Study flow chart. BID, twice daily; EOD, every other day; JAK, Janus kinase.

RuNiC is a phase Ib/II, open‐label, multicenter, dose‐finding study on ruxolitinib in combination with nilotinib (300 mg twice a day (BID)) and prednisone (50 mg every other day) for patients with MF [17, 18, 19]. The dose‐escalation phase (first six cycles, 28‐day cycles) initially planned with ruxolitinib starting from 10 mg BID was based on phase II studies [13], according to the classic (non‐Bayesian) model [20, 21]. Next, a dose‐expansion phase has been planned (12−24 cycles, 1−2 years), whereby an additional 10 patients in each arm (20 patients in total) will be treated according to their respective arm (Figure 1). For patients with prior ruxolitinib, no washout phase was needed.

A dose‐limiting toxicity (DLT) was defined as any treatment‐related toxicity occurring within the first 28 days (from Cycle 1 on Day 1 to Cycle 1 on Day 28) of treatment with ruxolitinib, nilotinib, and prednisone and that met any of the DLT criteria (Table S1).

Dose Modifications and dose delay of combination therapy: Hematologic toxicity is performed with ruxolitinib dose adjustment. This study does not contemplate a reduction in doses of nilotinib and prednisone except in cases of toxicities. In such cases, doses of nilotinib and prednisone will be reduced to 200 mg BID and 25 mg every other day (EOD), respectively. But for all patients participating in the study dose reductions of nilotinib are required for platelet count reductions to specified levels considered related (possibly, probably, definitely) to nilotinib.

### 2.3 Assessments

The primary objective was to determine the MTD and the RP3D of ruxolitinib in combination with nilotinib and prednisone. The secondary objectives included evaluating the clinical activity of ruxolitinib in combination with nilotinib and prednisone and the safety profile (the frequency and severity of AEs as graded by the National Cancer Institute CTCAE version 4.0) of this combination. The clinical activity was evaluated using the Eastern Oncology Cooperative Group (ECOG) performance status at Cycle 4 on Day 1, at Cycle 7 on Day 1, and at Cycle 12 on Day 28; the palpable spleen length reduction (the proportion of patients achieving a ≥50% reduction in palpable measurement), recorded from baseline to Cycle 7 on Day 1 and to Cycle 12 on Day 28; and the TSS and individual item scores in the modified MF‐symptom assessment form (SAF) [22], recorded at Cycle 4 on Day 1, at Cycle 7 on Day 1, and at Cycle 12 on Day 28. We have used the IWG‐MRT but using palpation assessment (which is referred to as 50%). A symptomatic response was defined as a reduction of 20% or more in TSS.

Chemosensitivity profiling was performed using PharmaFlow, as explained in Supplementary Methods. The mutational profiles were also determined by DNA from total bone marrow or peripheral blood samples. Targeted deep sequencing was performed as previously described using the Ion Torrent technology; we used a custom next generation sequencing (NGS) panel consisting of 43 genes recurrently mutated in myeloid diseases (Table S2) [23].

### 2.4 Statistical analysis

Quantitative variables are described with measures of central tendency and dispersion: mean, median, SD (standard deviation), Q1 (first quartile) and Q3 (third quartile), and minimum and maximum. Qualitative variables are described using absolute and relative frequencies (*N*, %). The description only concerned the descriptive analysis of the data. No statistical modeling procedures were performed. The analysis was performed using the IBM SPSS Statistics software, Version 22.0 (IBM Corp. Armonk, NY).

### 2.5 Ethical approvals

This study was approved by the institutional review board of the Hospital 12 de Octubre of Madrid (CEIm number 17/182) and the boards of the respective institutions before patient enrolment. The study was conducted following the principles of the Declaration of Helsinki. All the patients provided written informed consent. The trial has been registered at EudraCT: Number 2016‐005214‐21.

## 3 RESULTS

### 3.1 Study population

From November 2017 to June 2020, 21 patients were included in the study at six Spanish sites. Six patients failed the screening for participation and did not receive treatment; these patients were not included in the intention‐to‐treat or the per‐protocol populations. A total of 15 patients received at least one dose of ruxolitinib (Figure 1). Six patients completed the planned treatment (40%), and nine were withdrawn from the study because of disease progression, a lack of efficacy or no clinical benefit (*n* = 5), the withdrawal of consent (*n* = 2), an AE (pulmonary thromboembolism, *n* = 1), or the investigator's medical judgment (*n* = 1). Nine patients completed the escalation phase and seven patients the extension phase (Cycle 12): four patients continued with treatment after cycle 12.

#### 3.1.1 Demographic, clinical, and MF baseline characteristics

The demographic and clinical characteristics of the patients are described in Table 1.

**TABLE 1:** Baseline characteristics of patients.

Variable	Value
**Age**, median (range)—years	66.5 (53.4–70.8)
**Gender**—*n* (%)	
Male	9 (60)
Female	6 (40)
**Ethnicity**—*n* (%)	
Caucasian	14 (93.3)
Arabic	1 (6.7)
**Main comorbidities at baseline (> 10%)**—*n* (%)	
Cardiovascular diseases	6 (40)
Musculoskeletal disorders	5 (33.3)
Neurologic or psychiatric disorders	3 (20)
Gastrointestinal disorders	2 (13.3)
Endocrine disorders	2 (13.3)
Other	8 (53.3)
**Patients with transfusion 12** **weeks prior screening**—*n* (%)	4 (26.7)
**Median peripheral‐blood blast count** (range)—%	4 (2‐9)
**Hematology,** median (range)	
Hemoglobin—g/dL	10.8 (9.2–11.7)
Platelet count—x10^9^/L	230 (92–467)
Absolute neutrophil count—x10^9^/L	7.6 (6.2–13.8)
White‐cell count—x10^9^/L	10.6 (4.61–13.5)
**Median spleen length** (range)—cm	8 (6–20)
**ECOG performance status**—n(%): 0/1/2	5 (33.3)/7 (46.7)/7 (46.7)
**Time from diagnosis**, median (range)—years	2.91 (0.59–3.98)
**Type of myelofibrosis**—*n* (%)^a^	
Primary MF	6 (40)
Post‐polycythemia vera MF	4 (26.7)
Post‐essential thrombocythemia MF	4 (26.7)
**IPSS risk category**—*n* (%)^b^	
Low	1 (6.7)
Intermediate 1	5 (33.3)
Intermediate 2	4 (26.7)
High	1 (6.7)
**DIPSS risk category**—*n* (%)^c^	
Intermediate 1	7 (46.7)
Intermediate 2	6 (40)
High	5 (33.3)
**DIPSS‐Plus risk category**—*n* (%)^d^	
Intermediate 1	5 (33.3)
Intermediate 2	4 (26.7)
**Mutations at diagnosis**—*n* (%)	
JAK2 V617F	9 (60)
*CALR* Type 1	4 (26.7)
*MPL* W515L/K	1 (6.7)
**Patients with prior antineoplastic therapy**—*n* (%)	13 (86.7)
**Antineoplastic therapy type**—*n* (%)	
Hydroxyurea	11 (84.6)
Ruxolitinib	10 (76.9)
Other	5 (38.5)
Alpha‐ Interferon	2 (15.4)
Anagrelide	1 (7.7)
BKM120	1 (7.7)
Imetelstat	1 (7.7)
Melphalan	1 (7.7)
**Patients with prior antineoplastic radiotherapy**—*n* (%)	1 (6.7)

*Note*: ^a^Data were not available for one patient (6.7%). ^b^Data were not available for four patients (26.7%). ^c^Data were not available for three patients (20%). ^d^Data were not available for 6 patients (40%). DIPSS = Dynamic International Prognostic Scoring System; DIPSS‐Plus = Dynamic International Prognostic Scoring System Plus; ECOG = Eastern Cooperative Oncology Group. *CALR* = Calreticulin. **^e^** Data were not available for seven patients (46.7%). **^f^** Data were not available for 6 patients (40%); **^g^** Data were not available for 10 patients (66.7%). IPSS = International Prognostic Scoring System; JAK2 = Janus Kinase 2; MF = Myelofibrosis; *MPL* = Myeloproliferative leukemia virus gene; PV/TE = Polycythemia vera / Essential thrombocythemia; smPCR = single‐molecule PCR *TET2* = Tet methylcytosine diosygenase 2.

Most of the patients (*n* = 13, 86.7%) had received prior therapy for MF, including hydroxyurea and ruxolitinib in 11 patients (84.6%) and 10 patients (76.9%), respectively.

#### 3.1.2 Study treatment dosing

The maximum tolerated doses were 20 mg twice a day (BID) of ruxolitinib, 300 mg BID of nilotinib, and 50 mg EOD of prednisone, at Cycle 12.

The dose and duration for each treatment were analyzed for study Cycles 4, 7, and 12. A descriptive analysis of the different drug doses for the total number of patients in each cycle is provided in Tables S2 and S3. Dose reduction was performed in the following cases: for prednisone, in 6/12 cases to Cycle 4, 5/8 cases to Cycle 7, and 4/7 cases to Cycle 12; for nilotinib, in 4/12 cases to Cycle 4, 3/8 cases to Cycle 7, and 2/7 cases to Cycle 12; for ruxolitinib, in 4/12 cases to Cycle 4, 3/8 cases to Cycle 7, and 3/7 cases to Cycle 12.

The median (range) follow‐up duration, from the first treatment dose to the last monitoring data collected, was 401 (59−727) days.

### 3.2 Safety

All the patients experienced at least one AE throughout the study. Fourteen patients (93.3%) registered at least one AE related to any of the study treatments.

A summary of the main all‐grade AEs and grade 3−4 AEs is shown in Table 2. The most common AEs were asthenia (*n* = 10 patients, 66.7%); hyperglycemia (*n* = 5, 33.3%); thrombocytopenia (*n* = 4, 26.7%); anemia (*n* = 3, 20%); dyspnea (*n* = 3, 20%); insomnia (*n* = 3, 20%); and upper respiratory infection (*n* = 3, 20%). Grade 3−4 toxicity in ≥10% of the patients was only observed for hyperglycemia (*n* = 2, 13.3%); thrombocytopenia (*n* = 2, 13.3%); and anemia (*n* = 2, 13.3%).

**TABLE 2:** Adverse events.

AE	Any grade, *n* (%)	Grade ≥3, *n* (%)
Asthenia	10 (66.7)	1 (6.7)
Hyperglycemia	5 (33.3)	2 (13.3)
Thrombocytopenia	4 (26.7)	2 (13.3)
Anemia	3 (20.0)	2 (13.3)
Dyspnea	3 (20.0)	0 (0.0)
Insomnia	3 (20.0)	0 (0.0)
Nausea	3 (20.0)	0 (0.0)
Upper respiratory infection	3 (20.0)	0 (0.0)
ALT increased	2 (13.3)	1 (6.7)
Anxiety	2 (13.3)	0 (0.0)
AST increased	2 (13.3)	0 (0.0)
Constipation	2 (13.3)	0 (0.0)
Diarrea	2 (13.3)	0 (0.0)
Dizziness	2 (13.3)	0 (0.0)
Edemas	2 (13.3)	1 (6.7)
Fever	2 (13.3)	0 (0.0)
Hyporexia	2 (13.3)	0 (0.0)
Impaired renal function	2 (13.3)	0 (0.0)
Lower back pain	2 (13.3)	0 (0.0)
Slight fever	2 (13.3)	0 (0.0)
Vomit	2 (13.3)	0 (0.0)
Worsening of anemia	2 (13.3)	1 (6.7)
Bilateral pleural effusion	1 (6.7)	1 (6.7)
Cholestasis	1 (6.7)	1 (6.7)
Congestive heart failure	1 (6.7)	1 (6.7)
Decompensation of biventricular heart failure of multifactorial etiology	1 (6.7)	1 (6.7)
GGT increased	1 (6.7)	1 (6.7)
Lipase increased	1 (6.7)	1 (6.7)
Pericardial effusion	1 (6.7)	1 (6.7)
Pleural effussion	1 (6.7)	1 (6.7)
Pulmonary hypertension	1 (6.7)	1 (6.7)
Pulmonary tromboembolism	1 (6.7)	1 (6.7)
Worsening of thrombocytopenia	1 (6.7)	1 (6.7)

Abbreviations: AE, adverse event; ALT, alanine aminotransferase; AST, aspartate aminotransferase; GGT, gamma‐glutamyltransferase.

Overall, 251 AEs were reported, among which 90 were treatment‐related AEs (Table S4). The most common treatment‐related AEs (≥5% of the total) were hyperglycemia (22.2%), thrombocytopenia (13.3%), anemia (8.9%), and elevated ALT (6.7%). Temporary interruption of treatment occurred for five (33.3%) patients; dose adjustment was reported for one (6.7%) patient, and permanent discontinuation of the treatment was registered for three (20%) patients.

From the start of the study, a total of five treatment‐related SAEs were reported by two patients (13.3%): one patient had pericardial effusion and bilateral pleural effusion (both conditions related to nilotinib), and one patient had congestive heart failure, pleural effusion, and pulmonary hypertension (all conditions related to nilotinib). No deaths were registered throughout the study.

For the patients who discontinued due to nilotinib toxicity, both passed away 9 and 5 months later due to progression of the disease and splenic radiotherapy complications.

### 3.3 Clinical activity of ruxolitinib in combination with nilotinib and prednisone

**FIGURE 2:**
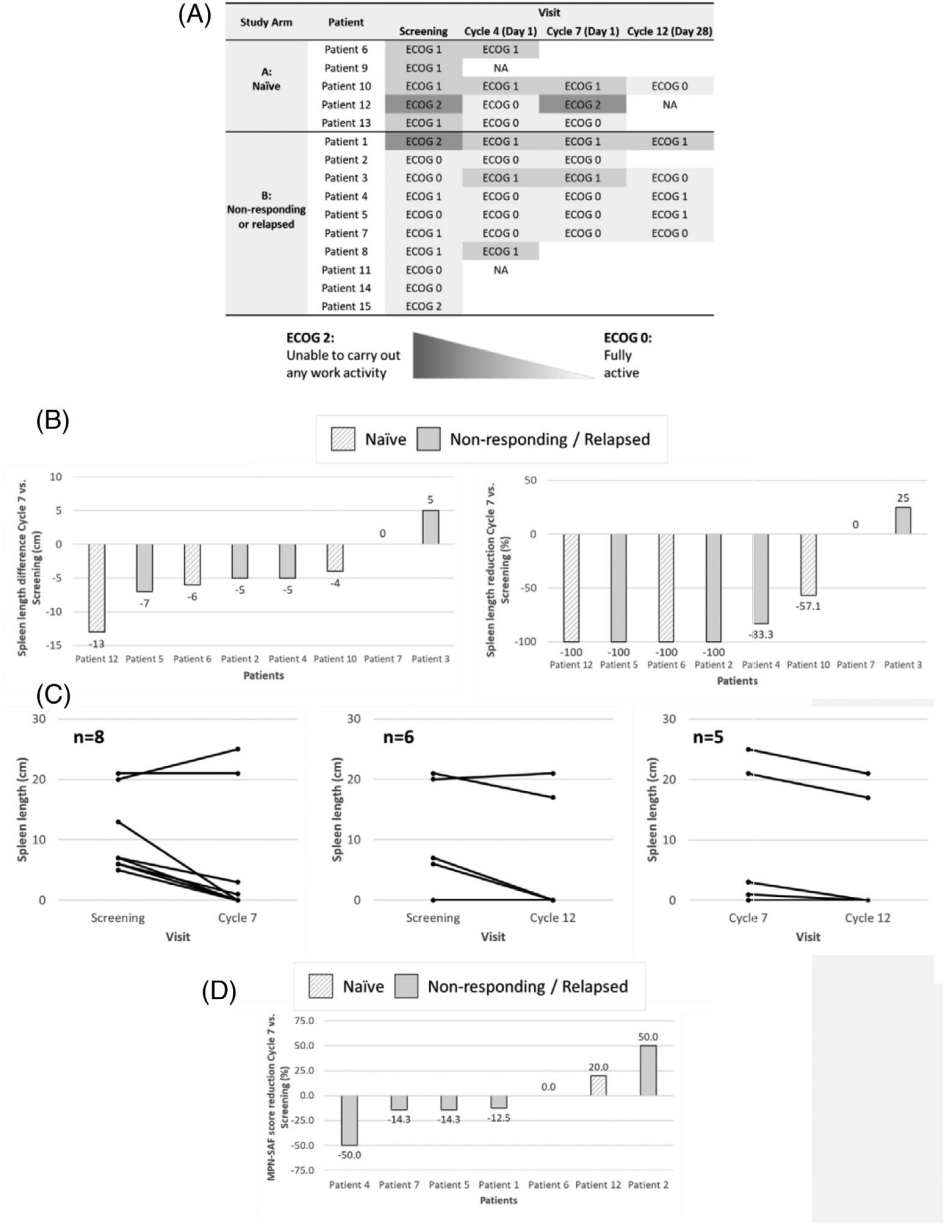
Clinical activity of ruxolitinib in combination with nilotinib and prednisone: (**A**) distribution of patients’ ECOG performance status scores, with all the visits considered; (**B**) change in spleen volume from baseline to end of Cycle 7; (**C**) comparison of the proportion of patients with each percentage decrease in palpable spleen length from screening to the end of Cycle 12; (**D)** change in MF‐SAF total symptom score from baseline to the end of Cycle 7. ECOG, Eastern Oncology Cooperative Group; MF‐SAF, Myelofibrosis Symptom Assessment Form; NA, not available.

The clinical activity is shown in Figure 2. Different distributions of patients were determined among the several ECOG performance status scores at each of the time points analyzed (Figure 2A). At screening, most patients were distributed between ECOG performance status scores of 1 and 2, while at Cycles 4, 7, and 12, most patients were distributed between ECOG performance status scores of 0 and 1. An improvement in ECOG performance status was observed at Cycle 4 in two of the five patients without pretreatment and in three of the ten patients who were resistant to pretreatment.

#### 3.3.1 Spleen length reduction

The spleen reduction is shown in Figure 2B,C. The Figure 2B,C includes separately the splenic response and the response to symptoms based on naive cases or after failure to ruxolitinib. Of the initial 15 patients, only eight had spleen lengths recorded at screening and Cycle 7. Four of 15 (27%) patients experienced a 100% spleen reduction at Cycle 7, and two additional patients achieved a >50% spleen reduction, accounting for an overall response rate of 40% at Cycle 7, assuming that the patients with no spleen measurements were non‐responders. Only five of the eight cases evaluated at Cycle 7 were evaluated for spleen length at Cycle 12, and among these, three patients experienced a 5% reduction in spleen length between Cycles 7 and 12. The mean (SD) and median (range) duration of response, estimated taking into consideration the spleen length reduction, were determined to be 8.4 (3.05) months and 8.38 (5.47, 11.53) months, respectively.

#### 3.3.2 MF‐SAF

The descriptive analysis of each MF‐SAF individual item and total score at screening and Cycles 4, 7, and 12, are shown in Table 3. As observed, the individual symptom values of the MF‐SAF median scores improved from screening to Cycles 4, 7, and 12, for most of the items. An increase in symptoms related to concentration difficulties and itching was observed at Cycle 12. From the initial 15 patients, only six had symptoms evaluated at screening and Cycle 7 (Figure 2D). Among these six patients, four patients experienced a total symptom score reduction (one patient reported a 50% reduction, two patients reported 14.3% reductions, and one patient reported a 12.5% reduction) from baseline to the end of Cycle 7. By contrast, one patient had the same total symptom score, and two patients reported increases (20% and 50%, respectively).

**TABLE 3:** Median MPN‐SAF TSS items and total score, at screening and cycles 4, 7 and 12.

	Median (range)			
MPN‐SAF‐ TSS (Items)	Screening (*n* = 15)	Cycle 4 (Day 1) (*n* = 9)	Cycle 7 (Day 1) (*n* = 8)	Cycle 12 (Day 28) (*n* = 5)
Fatigue	7.0 (5.0–9.0)	6.0 (5.0–8.0)	6.0 (5.0–7.0)	5.0 (3.5–8.5)
Early satiety	5.0 (0.0–7.0)	4.0 (0.0–5.0)	4.0 (0.8–4.8)	4.0 (1.0–4.5)
Abdominal discomfort	5.0 (0.0–8.0)	0.0 (0.0–4.0)	0.5 (0.0–4.3)	1.0 (0.04.5)
Inactivity	5.0 (1.0–8.0)	3.0 (0.0–6.0)	1.0 (0.0–2.8)	1.0 (0.5–6.0)
Concentration difficulties	2.0 (0.0–8.0)	3.0 (0.0–5.0)	1.0 (0.0–2.8)	2.0 (0.0–5.5)
Night sweats	5.0 (0.0–8.0)	0.0 (0.0–4.0)	3.0 (0.5–5.0)	1.0 (0.0–2.0)
Itching	4.0 (0.0–7.0)	2.0 (0.0–5.0)	1.0 (0.0–6.8)	3.0 (1.0–4.0)
Bone pain	4.0 (1.0–8.0)	2.5 (0.0–6.0)	3.0 (0.0–5.8)	1.0 (0.0–9.0)
Fever (>37°C)	0.0 (0.0–1.0)	0.0 (0.0–0.0)	0.0 (0.0–0.0)	0.0 (0.03.0)
Unintentional weight loss	1.0 (0.0–6.0)	0.0 (0.0–2.0)	0.0 (0.0–0.0)	0.0 (0.0–0.5)
Total score	40.0 (21.0–49.0)	24.0 (14.0–33.8)	23.0 (21.0–25.5)	17.0 (10.0–44.0)

Abbreviations: MFSAF‐TSS, myelofibrosis symptom assessment form total symptom score; SD, standard deviation.

#### 3.3.3 Ex vivo assays pharmaflow chemosensitivity profiling

We analyzed the correlations between the treatment with ruxolitinib in combination with nilotinib and prednisone by an ex vivo PharmaFlow study in samples from nine treated patients, and the patients’ clinical responses (Figure S1). This was feasible in eight out of the fifteen cases evaluated (53.3%). The ex vivo classification correlated with the clinical response to Cycle 7, since 75% of the samples from clinical responders were classified as sensitive. By contrast, 50% of the samples from patients who did not clinically respond to Cycle 7 were classified ex vivo resistant.

#### 3.3.4 Mutational profile by NGS

Mutational profiling was performed by NGS with a custom panel (Table S5) in 10 cases. Driver mutations were detected in the *JAK2* (seven cases, 46.7%), calreticulin (*CALR)* (two cases, 13.3%), and thrombopoietin (*MPL)* genes (one case, 6.7%). The mutational landscapes of the responders and nonresponders in Cycle 7 are shown in Figure S2. Among the seven cases with mutated *JAK2*, five cases were responders; both cases with mutated *CALR* were responders; and the only case with mutated *MPL* was a non‐responder. The most commonly co‐mutated genes were the *JAK2* gene with *BCORL1* (two cases) and *TET2* (two cases) and the *CALR* gene with *DNMT3A* (two cases) and *TET2* (three cases). The *MPL* gene co‐mutated with *BCORL1*, *DNMT3A*, *PHF6*, and *SH2B3* (one case). The distribution of the most frequent driver mutations in relation to the Cycle 7 response was as follows: among the four cases with mutated *TET2*, three were responders to Cycle 7, and among the four cases with mutated *BCORL1*, two were responders to Cycle 7.

## 4 DISCUSSION

The main objectives of this phase Ib/II trial were to determine the MTD, toxicity, clinical efficacy, and biological activity of ruxolitinib in combination with nilotinib and prednisone in MF patients, naïve or previously treated with JAK inhibitors. This combination was safe and tolerable with relevant clinical activity in these patients.

In phase III ruxolitinib clinical trials, the rate of discontinuation of ruxolitinib treatment because of AEs was 11% (as compared with 10.6% in the placebo group) [9], with anemia (43%) and thrombocytopenia (17%) being the most common AEs. In our study, 14 patients registered at least one treatment‐related AE (the most common were hyperglycemia [22.2%], thrombocytopenia [13.3%], and anemia [8.9%]) but grade 3−4 toxicity was only seen for hyperglycemia (*n* = 2, 13.3%); thrombocytopenia (*n* = 2, 13.3%); and anemia (*n* = 2, 13.3%). These results led to the permanent discontinuation of the treatment in three (20%) patients. In general, the doses of the different drugs in the combination received were well adjusted to what was expected, and there were no important dose losses due to delays, interruptions, or reductions. Thus, the results show that the AEs with this combination were manageable and in line with those reported for ruxolitinib.

The preliminary efficacy data show that most of the patients shifted from ECOG performance status scores of 1 and 2 at the screening to ECOG performance status scores of 0 and 1 at Cycles 4, 7, and 12. Most of the patients had a ≥25% reduction in palpable spleen size while receiving the study treatment. In addition, in most of the MPN‐SAF score items, the median individual symptom values improved from screening to Cycles 4, 7, and 12.

The so‐called “add‐on” approach (by adding a targeted agent to ruxolitinib for synergy) is currently attracting interest for patients showing insufficient responses to ruxolitinib monotherapy [24, 25, 26, 27, 28]. Since MF is a complex disease in which tyrosine‐kinase‐mediated signaling pathways are involved in the production of hematopoietic clones and with the fibrosis of the bone marrow, the use of drugs in combination against different targets may be beneficial. Previous data have shown that ruxolitinib combined with prednisone and nilotinib exhibits synergistic effects in human cell lines and primary cells from myeloproliferative neoplasms (MPN) [29]. Ruxolitinib showed the ability to diminish or stabilize fibrosis. Ruxolitinib plus nilotinib showed a synergistic behavior that blocked colony formation in patients’ primary cells and inhibited the phosphorylation of STAT5 and ERK1/2. In addition, the combination with prednisone increased this synergy and inhibited the synthesis of collagen in bone marrow mesenchymal cells [29].

Moreover, ex vivo PharmaFlow analyses in samples of patients treated with the combination showed a trend of correlations between the sensitive ex vivo classification and clinical responses, but a larger sample is needed to confirm these results. Regarding the influence of the mutational profile and the response at Cycle 7, five of the seven cases with a mutated *JAK2* gene and the two cases with a mutated *CALR* gene were determined to be responders. On the contrary, the only case with a mutated *MPL* gene was a non‐responder. Although the series is small, the mutated genes associated especially with the nonresponder group were *PHF6*, *SRSF2*, *SH2B3*, and *ZRSR2*, and we observed no difference in response between JAK2 and CALR patients.

The responses to ruxolitinib treatment are known to be transitory. Ruxolitinib rechallenge has shown limited effectiveness after a period of treatment interruption [30, 31]. *JAK2‐V617F* leukemia cell lines could be re‐sensitized to ruxolitinib after a period of withdrawal [31]. Nine of thirteen MF patients retreated with ruxolitinib after the loss of the initial response or an inadequate response registered significant spleen size reductions, and twelve of thirteen patients reported symptom improvement [30]. In this case series, ruxolitinib was ultimately discontinued in seven patients due to a loss of response, inclusion in another clinical trial, or intolerance to the treatment [30].

The main limitation of our study is that, due to the number of patients finally enrolled, it was not possible to comply with the evaluation characteristics of the MTD, and therefore, no data regarding the main objective of the trial were obtained. Another limitation is that seven intermediate‐1‐risk patients (46.7%) were included, making it difficult to assess the efficacy as compared with other studies. No differences in response between naïve and pretreated patients could be observed, probably due to the low number of patients. However, our study provides relevant information on the efficacy and safety of the combination, its correlations with ex vivo data, and the mutational landscape of the patients.

In conclusion, the current results provide evidence that ruxolitinib in combination with nilotinib and prednisone has therapeutic activity in MF, with a good safety profile.

## AUTHOR CONTRIBUTIONS

*Conceptualization, methodology, formal analysis, investigation, resources, writing‐ original draft, and funding acquisition*: Rosa Ayala, Rafael Alonso, and Joaquin Martinez‐Lopez. *Investigation and writing‐review*: Valentín García‐Gutiérrez, Alberto Alvarez‐Larrán, Santiago Osorio, Jose M. Sánchez‐Pina, María Teresa Gómez‐Casares, Antonia Duran, and Juan Carlos Hernandez‐Boluda. *Formal analysis, data curation, and writing‐ review*: Noemi Alvarez, Gonzalo Carreño, and Julian Gorrochategi.

## CONFLICT OF INTEREST STATEMENT

J.M‐L. declares honoraria for lectures; membership on advisory boards with Janssen, BMS, Sanofi, Novartis, Incyte, Roche, and Amgen; and membership on the boards of directors of Hosea and Altum Sequencing. R.A. is an advisory board member for Novartis and Incyte; received speaker honoraria from Astellas, Celgene, and Novartis; and is a member on the board of directors of Altum Sequencing. The remaining authors declare no competing financial interests.

## Supporting information

Supporting Information

Supporting Information

## Data Availability

Questions regarding data sharing should be addressed to the corresponding author. For original data and study protocol, please contact Prof. Joaquín Martinez‐Lopez at jmarti01@med.ucm.es. Deidentified individual participant data that underlie the reported results will be made available 3 months after publication for a period of 5 years after the publication date.

## References

[1] Tefferi A , Guglielmelli P , Pardanani A , Vannucchi AM . Myelofibrosis treatment algorithm 2018. Blood Cancer J. 2018;8(8):72. 30065290 10.1038/s41408-018-0109-0 PMC6068139

[2] Tefferi A , Nicolosi M , Mudireddy M , Szuber N , Finke CM , Lasho TL , et al. Driver mutations and prognosis in primary myelofibrosis: Mayo‐Careggi MPN alliance study of 1,095 patients. Am J Hematol. 2018;93(3):348–55. 29164670 10.1002/ajh.24978

[3] Quintás‐Cardama A , Vaddi K , Liu P , Manshouri T , Li J , Scherle PA , et al. Preclinical characterization of the selective JAK1/2 inhibitor INCB018424: therapeutic implications for the treatment of myeloproliferative neoplasms. Blood. 2010;115(15):3109–17. 20130243 10.1182/blood-2009-04-214957 PMC3953826

[4] EMA . Summary of product characteristics Jakavi (5 mg /10 mg /15 mg / 20 mg tablets). EMA: European Medicines Agency; ema.europa.eu 2021.

[5] Cervantes F , Vannucchi AM , Kiladjian JJ , Al‐Ali HK , Sirulnik A , Stalbovskaya V , et al. Three‐year efficacy, safety, and survival findings from COMFORT‐II, a phase 3 study comparing ruxolitinib with best available therapy for myelofibrosis. Blood. 2013;122(25):4047–53. 24174625 10.1182/blood-2013-02-485888

[6] McMullin MF , Harrison CN , Niederwieser D , Demuynck H , Jäkel N , Gopalakrishna P , et al. The use of erythropoiesis‐stimulating agents with ruxolitinib in patients with myelofibrosis in COMFORT‐II: an open‐label, phase 3 study assessing efficacy and safety of ruxolitinib versus best available therapy in the treatment of myelofibrosis. Exp Hematol Oncol. 2015;4(26):015–21. 10.1186/s40164-015-0021-2 PMC4570722 26380150

[7] Vannucchi AM , Kantarjian HM , Kiladjian JJ , Gotlib J , Cervantes F , Mesa RA , et al. A pooled analysis of overall survival in COMFORT‐I and COMFORT‐II, 2 randomized phase III trials of ruxolitinib for the treatment of myelofibrosis. Haematologica. 2015;100(9):1139–45. 26069290 10.3324/haematol.2014.119545 PMC4800694

[8] Verstovsek S , Mesa RA , Gotlib J , Gupta V , DiPersio JF , Catalano JV , et al. Long‐term treatment with ruxolitinib for patients with myelofibrosis: 5‐year update from the randomized, double‐blind, placebo‐controlled, phase 3 COMFORT‐I trial. J Hematol Oncol. 2017;10(1):55. 28228106 10.1186/s13045-017-0417-z PMC5322633

[9] Verstovsek S , Mesa RA , Gotlib J , Levy RS , Gupta V , DiPersio JF , et al. A double‐blind, placebo‐controlled trial of ruxolitinib for myelofibrosis. N Engl J Med. 2012;366(9):799–807. 22375971 10.1056/NEJMoa1110557 PMC4822164

[10] Verstovsek S , Mesa RA , Gotlib J , Levy RS , Gupta V , DiPersio JF , et al. Efficacy, safety and survival with ruxolitinib in patients with myelofibrosis: results of a median 2‐year follow‐up of COMFORT‐I. Haematologica. 2013;98(12):1865–71. 24038026 10.3324/haematol.2013.092155 PMC3856961

[11] Verstovsek S , Mesa RA , Gotlib J , Levy RS , Gupta V , DiPersio JF , et al. Efficacy, safety, and survival with ruxolitinib in patients with myelofibrosis: results of a median 3‐year follow‐up of COMFORT‐I. Haematologica. 2015;100(4):479–88. 25616577 10.3324/haematol.2014.115840 PMC4380721

[12] Arenas A , Hernandez‐Campo P , Gorrochategui J , Primo D , Ayala RM , Ferrer‐Lores B , et al. Ruxolitinib in combination with nilotinib and prednisolone, a new synergistic approach to treat myelofibrosis. Blood. 2014;124(21):903.

[13] Gallipoli P , Cook A , Rhodes S , Hopcroft L , Wheadon H , Whetton AD , et al. JAK2/STAT5 inhibition by nilotinib with ruxolitinib contributes to the elimination of CML CD34+ cells in vitro and in vivo. Blood. 2014;124(9):1492–501. 24957147 10.1182/blood-2013-12-545640 PMC4148771

[14] Rhee CK , Lee SH , Yoon HK , Kim SC , Lee SY , Kwon SS , et al. Effect of nilotinib on bleomycin‐induced acute lung injury and pulmonary fibrosis in mice. Respiration. 2011;82(3):273–87. 21659722 10.1159/000327719 PMC7068797

[15] Shaker ME , Shiha GE , Ibrahim TM . Comparison of early treatment with low doses of nilotinib, imatinib and a clinically relevant dose of silymarin in thioacetamide‐induced liver fibrosis. Eur J Pharmacol. 2011;670(2‐3):593–600. 21925495 10.1016/j.ejphar.2011.08.041

[16] Shaker ME , Zalata KR , Mehal WZ , Shiha GE , Ibrahim TM . Comparison of imatinib, nilotinib and silymarin in the treatment of carbon tetrachloride‐induced hepatic oxidative stress, injury and fibrosis. Toxicol Appl Pharmacol. 2011;252(2):165–75. 21316382 10.1016/j.taap.2011.02.004 PMC3895503

[17] Barraco D , Maffioli M , Passamonti F . Standard care and investigational drugs in the treatment of myelofibrosis. Drugs Context. 2019;8:212603. 31645880 10.7573/dic.212603 PMC6788389

[18] Hochhaus A , Baccarani M , Silver RT , Schiffer C , Apperley JF , Cervantes F , et al. European LeukemiaNet 2020 recommendations for treating chronic myeloid leukemia. Leukemia. 2020;34(4):966–84. 32127639 10.1038/s41375-020-0776-2 PMC7214240

[19] Saglio G , Kim DW , Issaragrisil S , le Coutre P , Etienne G , Lobo C , et al. Nilotinib versus imatinib for newly diagnosed chronic myeloid leukemia. N Engl J Med. 2010;362(24):2251–9. 20525993 10.1056/NEJMoa0912614

[20] Le Tourneau C , Lee JJ , Siu LL . Dose escalation methods in phase I cancer clinical trials. J Natl Cancer Inst. 2009;101(10):708–20. 19436029 10.1093/jnci/djp079 PMC2684552

[21] Storer BE . Design and analysis of phase I clinical trials. Biometrics. 1989;45(3):925–37. 2790129

[22] Mesa RA , Schwager S , Radia D , Cheville A , Hussein K , Niblack J , et al. The Myelofibrosis Symptom Assessment Form (MFSAF): an evidence‐based brief inventory to measure quality of life and symptomatic response to treatment in myelofibrosis. Leuk Res. 2009;33(9):1199–203. 19250674 10.1016/j.leukres.2009.01.035 PMC4419687

[23] Ayala R , Rapado I , Onecha E , Martínez‐Cuadrón D , Carreño‐Tarragona G , Bergua J , et al. The mutational landscape of acute myeloid leukaemia predicts responses and outcomes in elderly patients from the PETHEMA‐FLUGAZA phase 3 clinical trial. Cancers (Basel). 2021;13(10):2458. 34070172 10.3390/cancers13102458 PMC8158477

[24] Stivala S , Codilupi T , Brkic S , Baerenwaldt A , Ghosh N , Hao‐Shen H , et al. Targeting compensatory MEK/ERK activation increases JAK inhibitor efficacy in myeloproliferative neoplasms. J Clin Invest. 2019;129(4):1596–611. 30730307 10.1172/JCI98785 PMC6436863

[25] Nieborowska‐Skorska M , Maifrede S , Dasgupta Y , Sullivan K , Flis S , Le BV , et al. Ruxolitinib‐induced defects in DNA repair cause sensitivity to PARP inhibitors in myeloproliferative neoplasms. Blood. 2017;130(26):2848–59. 29042365 10.1182/blood-2017-05-784942 PMC5746670

[26] Fisher DAC , Miner CA , Engle EK , Hu H , Collins TB , Zhou A , et al. Cytokine production in myelofibrosis exhibits differential responsiveness to JAK‐STAT, MAP kinase, and NFκB signaling. Leukemia. 2019;33(8):1978–95. 30718771 10.1038/s41375-019-0379-y PMC6813809

[27] Fisher DAC , Malkova O , Engle EK , Miner CA , Fulbright MC , Behbehani GK , et al. Mass cytometry analysis reveals hyperactive NF Kappa B signaling in myelofibrosis and secondary acute myeloid leukemia. Leukemia. 2017;31(9):1962–74. 28008177 10.1038/leu.2016.377 PMC5540814

[28] Bhagwat N , Koppikar P , Keller M , Marubayashi S , Shank K , Rampal R , et al. Improved targeting of JAK2 leads to increased therapeutic efficacy in myeloproliferative neoplasms. Blood. 2014;123(13):2075–83. 24470592 10.1182/blood-2014-01-547760 PMC3968390

[29] Cortés AA , Diaz RA , Hernández‐Campo P , Gorrochategui J , Primo D , Robles A , et al. Ruxolitinib in combination with prednisone and nilotinib exhibit synergistic effects in human cells lines and primary cells from myeloproliferative neoplasms. Haematologica. 2019;104(5):937–46. 30545926 10.3324/haematol.2018.201038 PMC6518898

[30] Gerds A , Su D , Martynova A , Pannell B , Mukherjee S , O'Neill C , et al. Ruxolitinib rechallenge can improve constitutional symptoms and splenomegaly in patients with myelofibrosis: a case series. Clin Lymphoma Myeloma Leuk. 2018;18(11):e463–e8. 30115545 10.1016/j.clml.2018.06.025

[31] Koppikar P , Bhagwat N , Kilpivaara O , Manshouri T , Adli M , Hricik T , et al. Heterodimeric JAK‐STAT activation as a mechanism of persistence to JAK2 inhibitor therapy. Nature. 2012;489(7414):155–9. 22820254 10.1038/nature11303 PMC3991463

