# Supplementary material for: Janus kinase inhibitor ruxolitinib in combination with nilotinib and prednisone in patients with myelofibrosis (RuNiC study): A phase Ib, multicenter study
Source: EJHaem. 2023 Apr 16;4(2):401–9. doi: 10.1002/jha2.685 (PMC10188506; doi:10.1002/jha2.685)
Supplement: Supplementary file 1 — Supporting Information [file JHA2-4-401-s002.docx]

# Janus kinase inhibitor ruxolitinib in combination with nilotinib and prednisone in patients with myelofibrosis (RuNiC study): a phase Ib, multicentre study.

### Authors.

1Rosa Ayala, 2Rafael Alonso Fernández, 4Valentín García-Gutiérrez, 5Alberto Alvarez-Larrán,

6Santiago Osorio, 2Jose M. Sánchez-Pina, 2Gonzalo Carreño, 3Noemi Álvarez, 7María

Teresa Gómez-Casares, 8Antonia Duran, 9Julian Gorrochategi, 10Juan Carlos Hernández-Boluda and 1Joaquín Martínez-López

### Affiliations.

1Haematological Malignancies Clinical Research Unit, Hospital Universitario 12 de Octubre, Universidad Complutense, CNIO, CIBERONC, Madrid, Spain. 2Hematology Department, Hospital Universitario 12 de Octubre, Madrid, Spain. 3Department of Translational Hematology, Research Institute Hospital 12 de Octubre (i+12), Madrid, Spain 4Hematology Department, Hospital Universitario Ramón y Cajal, Madrid, Spain. 5Hematology Department, Hospital ClíNic, Barcelona, Spain; 6Hematology Department Hospital General U. Gregorio Marañón, Madrid, Spain; 7Hematology Department Hospital Universitario de Gran Canaria Dr. Negrin, Las Palmas de Gran Canaria, Spain; 8Hematology Department Hospital Universitario Son Espases, Palma de Mallorca, Spain; 9Vivia Biotech, S.L. Tres Cantos, Madrid, Spain; 10Hospital Clínico Universitario- INCLIVA, Valencia, Spain.

### SUPPLEMENTARY METHODS


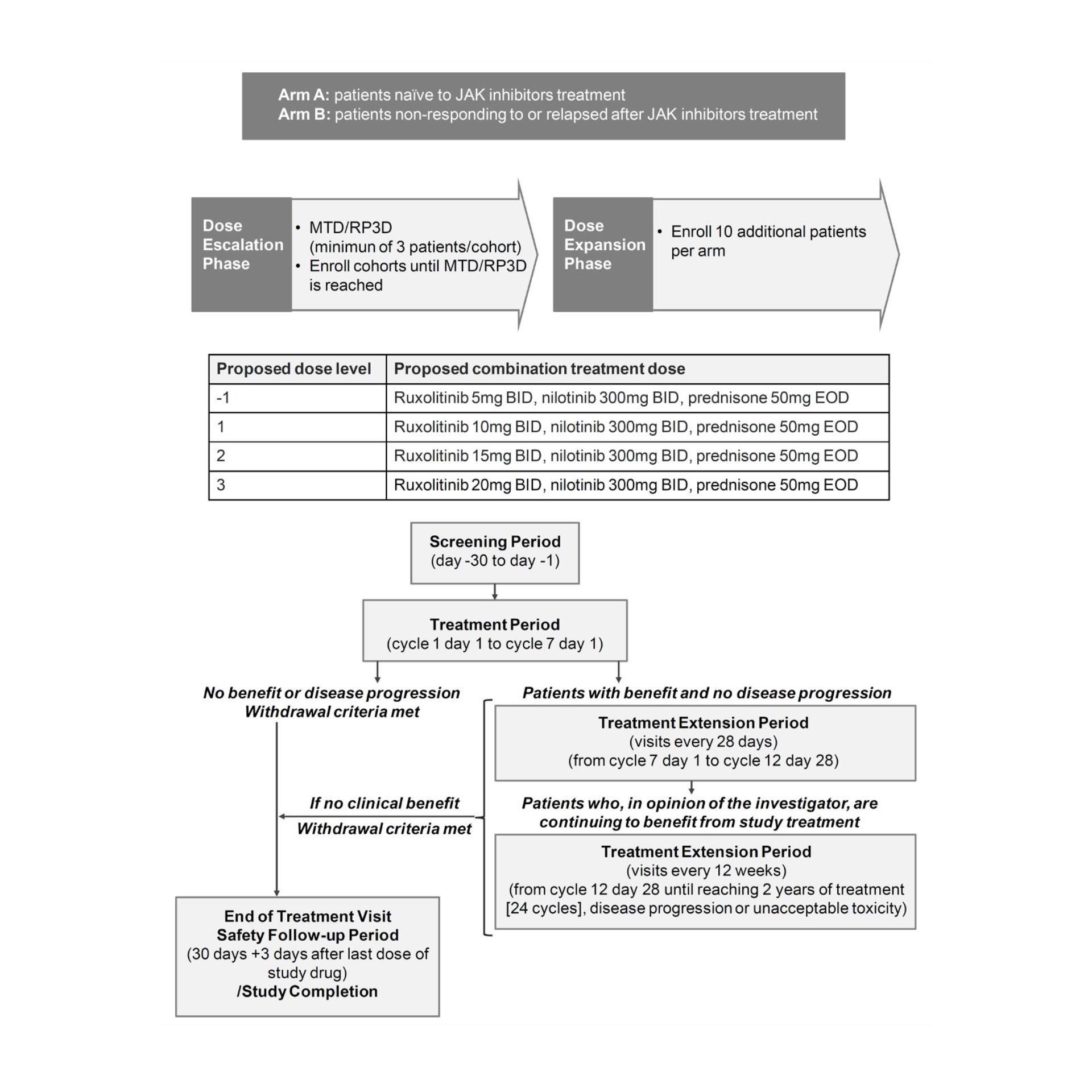


### Supplementary Figure 1: Planned study design.

Abbreviations: BID =Twice a day; EOD = Every other day; JAK = Janus Kinase; MTD = Maximum tolerated dose; RP3D= Recommended phase III dose.

The key inclusion criteria were: i) age ≥18 years; ii) diagnosis of primary MF, post-polycythemia vera MF, or post-essential thrombocythemia MF and at least intermediate-risk level-1, according to the International Working Group1; iii) had a palpable spleen (≥5 cm from the costal margin); iv) active symptoms of MF as measured by Total Symptom Score [TSS] and individual item scores in the modified Myelofibrosis Symptom Assessment Form [MF-SAF]2 (minimum of 5 points in at least one item, or two items of at least 3 points); and v) platelet count ≥50×109/L, absolute neutrophil count >1x109/L, , and peripheral blood blast count of <5%, criteria at screening or cycle 1 (day 1). An Eastern Oncology Cooperative Group (ECOG) performance status of ≤2 was required at screening. Finally, patients had discontinued all drugs used to treat underlying MF disease no later than seven days before cycle 1 (day 1). The key exclusion criteria included: i) pregnancy; ii) previous treatment with JAK inhibitors that resulted in clinically significant toxicities; iii) stem cell transplantation eligibility; and iv) splenic irradiation within 12 months prior screening. The study consisted of two cohorts: patients naïve to JAK inhibitors treatment and non-responding to or relapsed after JAK inhibitors treatment. Patients non-responding to or relapsed after 12 weeks on JAK inhibitors treatment were defined as those with no improvement in spleen length; <25% spleen length reduction or ≥25% and <49% without symptomatic improvement; or lost of clinical benefit as per investigator. The starting dose of nilotinib was the standard dose used in the treatment of chronic myeloid leukemia. The dose of 50 mg for prednisone on alternate days was based on the use of long- term corticoids therapy in other hematological malignancies (myeloma multiple where no excess of infectious complications were detected).3 During the dose-escalation phase, the ruxolitinib dose was planned to range from 10 mg BID to 20 mg BID to be evaluated in combination with nilotinib and prednisone. In this phase, the combination treatment's maximum tolerated dose (MTD) was planned to be based upon the estimation of dose-limiting toxicity (DLT) in cycle 1 for patients in the dose-determining set. This was estimated by following classic (non-Bayesian) model4, 5.

**Supplementary Figure 2**


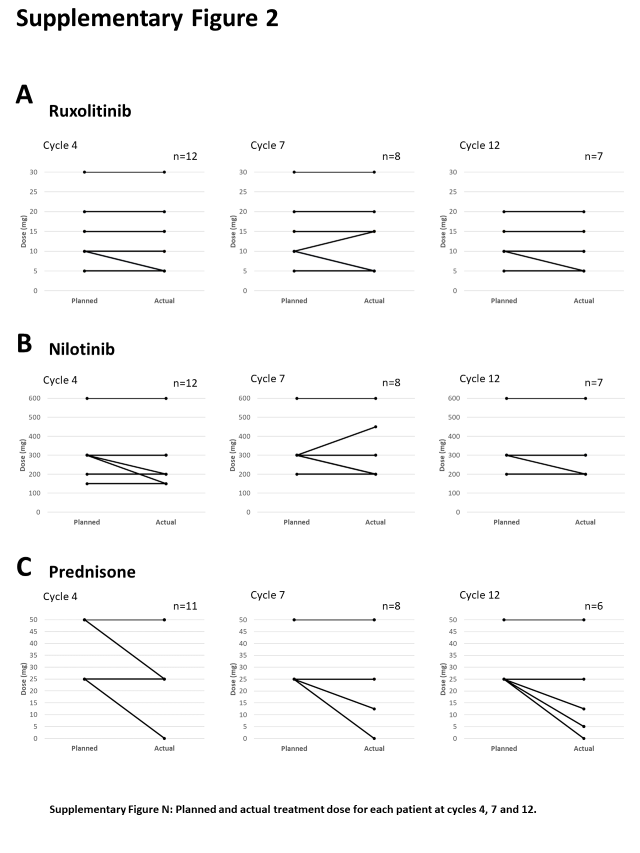


### Supplementary Figure 2: Planned and actual treatment dose for each patient at cycles 4, 7 and 12

Abbreviations: BID =Twice a day; EOD = Every other day; JAK = Janus Kinase; MTD = Maximum tolerated dose; RP3D= Recommended phase III dose.


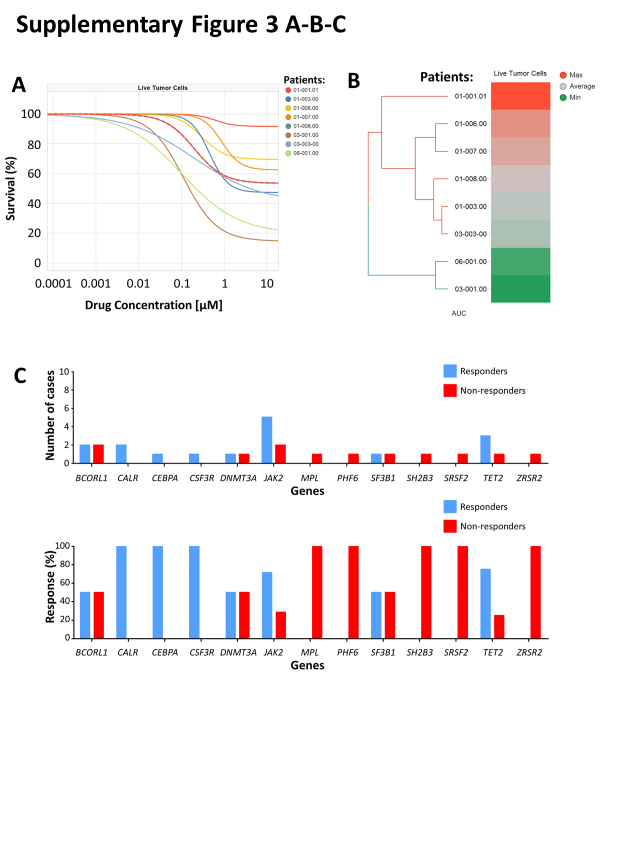


**Supplementary Figure 3A-B: PharmaFlow platform assays. (3A)** Evaluation of the cytotoxic effects of compounds with the depletion assay. Each coloured line represents a patient. The survival index (y-axis) ranges from 100% to 0% displaying the cell depletion after exposure to a drug concentration (x-axis). **(3B)** Headmap showing in color scale the area under the curve (AUC) value from doce-response curves as a marker of ex─vivo activity integrating potency and efficacy. Rows, corresponding to each sample were sorted and classified with the dendrogram and cluster analysis using Euclidian distance. We analyzed the correlations between the treatment with ruxolitinib in combination with nilotinib and prednisone by an ex vivo PharmaFlow study in samples from nine treated patients, and the patients’ clinical responses. This was feasible in eight out of the fifteen cases evaluated (53.3%). The ex vivo classification correlated with the clinical response to Cycle 7, since 75% of the samples from clinical responders were classified as sensitive. By contrast, 50% of the samples from patients who did not clinically respond to Cycle 7 were classified ex vivo resistant.

For ex vivo drug sensitivity assays, fresh bone marrow samples from 9 patients were collected. in heparinised tubes and analyzed by the processing laboratory within 24 hours from extraction. Sample incubation with the corresponding drugs was performed maintaining the native micro- environment as previously described.6, 7 After incubation for 48 hours, red blood cells were lysed and remaining cells were washed, labelled with fluorescent conjugated antibodies, and injected in the flow cytometer. Labelling was performed by Annexin-V fluorescein isothiocyanate and at least two of the best monoclonal antibodies that unequivocally identified the pathological cells according to EuroFlow panels.8, 9 The number of remaining live pathological cells (LPCs) after incubation with a varied drug concentrations for a given time period represented the response effect and served to calculate dose-response curves. Normalization of dose-response curves and potential spontaneous cell death were performed by control cells incubated under the same conditions but without drug exposure.

**Supplementary Figure 3C: The landscape of mutated genes detected by DNA sequencing Ion Torrent Technology, using a custom NGS panel consisting of 43 genes recurrently mutated in myeloid diseases.** Number of cases **(A)** and percentage **(B)** of responders (blue) and non-responders (red). Mutational profiling was performed via targeted NGS using a custom panel of 43 genes implicated in myeloid pathology in 10 cases. Driver mutations were detected in the JAK2 (seven cases, 46.7%), calreticulin (CALR) (two cases, 13.3%), and thrombopoietin (MPL) genes (one case, 6.7%). The mutational landscapes of the responders and non-responders in Cycle 7 are shown in Supplemental Figure S2C. Among the seven cases with mutated JAK2, five cases were responders; both cases with mutated CALR were responders; and the only case with mutated MPL was a non-responder. The most commonly co-mutated genes were the JAK2 gene with BCORL1 (two cases) and TET2 (two cases) and the CALR gene with DNMT3A (two cases) and TET2 (three cases). The MPL gene co-mutated with BCORL1, DNMT3A, PHF6, and SH2B3 (one case). The distribution of the most frequent driver mutations in relation to the Cycle 7 response were as follows: among the four cases with mutated TET2, three were responders to Cycle 7, and among the four cases with mutated BCORL1, two were responders to Cycle 7.

The total number of reads obtained in each sample was two million, with an average depth of coverage >2000 reads per nucleotide and high uniformity amongst all fragments (92%). Data analyses were performed using Ion Reporter v4.4 software (Life Technologies, Carlsbad, CA, USA), which identified single nucleotide variants (SNV) and small insertions or deletions (InDels). We employed Ion Reporter default parameters and filtered out variants with a total coverage of at least 70 reads and a variant allelic coverage of at least 10 reads. Variants with a minor allelic frequency >0.01 in the general population according to the single nucleotide polymorphism database (NCBI, dbSNP150) and/or the 5000-exome sequencing project were also rejected as possible polymorphisms (https://evs.gs.washington.edu/EVS; accessed on 20 December 2019). Filtered variants were then annotated using the Catalogue of Somatic Mutations in Cancer (COSMIC) database (https://cancer.sanger.ac.uk/census; accessed on 20 December 2019), allowing those variants present in some tumors to be retained, and those present in dbSNP and previously identified as cancer mutations to be retained. Filtered variants that were absent from dbSNP or COSMIC but were “deleterious” due to associated functional changes at the protein level, or due to their occurrence in conserved regions, were considered in the final analysis.
