# Supplementary material for: Janus kinase inhibitor ruxolitinib in combination with nilotinib and prednisone in patients with myelofibrosis (RuNiC study): A phase Ib, multicenter study
Source: EJHaem. 2023 Apr 16;4(2):401–9. doi: 10.1002/jha2.685 (PMC10188506; doi:10.1002/jha2.685)
Supplement: Supplementary file 2 — Supporting Information [file JHA2-4-401-s001.docx]

**Supplemental Table 3: Study treatment relative dose intensities, at cycles 4, 7 and 12.**

| **Treatment** | **Cycle** | **Relative dose intensity**  **median (range)** |
| --- | --- | --- |
| **Ruxolitinib** | **Cycle 4**  **(n=12)** | 1.04  (0.952-1.04) |
|  | **Cycle 7**  **(n=8)** | 1  (0.903-1.04) |
|  | **Cycle 12**  **(n=7)** | 1.02  (0.679-1.04) |
| **Nilotinib** | **Cycle 4**  **(n=12)** | 0.81  (0.207-1.08) |
|  | **Cycle 7**  **(n=8)** | 1.02  (0.759-1.04) |
|  | **Cycle 12**  **(n=7)** | 1.08  (1.04-1.17) |
| **Prednisone** | **Cycle 4**  **(n=11)** | 1.04  (0.952-1.04) |
|  | **Cycle 7**  **(n=8)** | 1  (0.697-1.04) |
|  | **Cycle 12**  **(n=6)** | 1.08  (1.04-1.17) |

**Supplementary table 2: Study treatment doses for each patient at cycles 4, 7 and 12.**

| **Variable** | | **Ruxolitinib** | | | **Nilotinib** | | | **Prednisone** | | |
| --- | --- | --- | --- | --- | --- | --- | --- | --- | --- | --- |
|  |  | **Cycle 4**  **(n=12)** | **Cycle 7**  **(n=8)** | **Cycle 12**  **(n=7)** | **Cycle 4**  **(n=12)** | **Cycle 7**  **(n=8)** | **Cycle 12**  **(n=7)** | **Cycle 4**  **(n=11)** | **Cycle 7**  **(n=8)** | **Cycle 12**  **(n=6)** |
| **Patient 1** | **Planned dose (mg)** | 10 | 10 | 10 | 300 | 300 | 300 | 50 | 50 | 50 |
|  | **Actual dose (mg)** | 5 | 5 | 5 | 300 | 450 | 300 | 25 | 25 | 5 |
|  | **Actual duration (days)** | 27 | 27 | 27 | 27 | 27 | 27 | 27 | 27 | 27 |
| **Patient 2** | **Planned dose (mg)** | 10 | 10 | ─ | 300 | 300 | ─ | 50 | 50 | ─ |
|  | **Actual dose (mg)** | 10 | 15 | ─ | 300 | 300 | ─ | 25 | 25 | ─ |
|  | **Actual duration (days)** | 27 | 27 | ─ | 27 | 27 | ─ | 27 | 27 | ─ |
| **Patient 3** | **Planned dose (mg)** | 10 | 10 | 10 | 300 | 300 | 300 | 50 | 50 | 50 |
|  | **Actual dose (mg)** | 5 | 5 | 10 | 150 | 200 | 300 | 25 | 12.5 | 12.5 |
|  | **Actual duration (days)** | 28 | 29 | 24 | 28 | 29 | 24 | 28 | 29 | 24 |
| **Patient 4** | **Planned dose (mg)** | 15 | 15 | 15 | 300 | 300 | 300 | 50 | 50 | 50 |
|  | **Actual dose (mg)** | 15 | 15 | 15 | 200 | 200 | 200 | 0 | 0 | 0 |
|  | **Actual duration (days)** | 27 | 29 | 27 | 27 | 29 | 27 | 27 | 29 | 27 |
| **Patient 5** | **Planned dose (mg)** | 20 | 20 | 20 | 300 | 300 | 300 | 50 | 50 | 50 |
|  | **Actual dose (mg)** | 20 | 20 | 20 | 300 | 300 | 300 | 25 | 25 | 25 |
|  | **Actual duration (days)** | 27 | 27 | 27 | 27 | 27 | 27 | 27 | 27 | 27 |
| **Patient 6** | **Planned dose (mg)** | 10 | ─ | ─ | 300 | ─ | ─ | 50 | ─ | ─ |
|  | **Actual dose (mg)** | 5 | ─ | ─ | 150 | ─ | ─ | 25 | ─ | ─ |
|  | **Actual duration (days)** | 27 | ─ | ─ | 27 | ─ | ─ | 25 | ─ | ─ |
| **Patient 7** | **Planned dose (mg)** | 15 | 15 | 15 | 300 | 300 | 300 | 50 | 50 | 50 |
|  | **Actual dose (mg)** | 15 | 15 | 5 | 300 | 300 | 300 | 50 | 50 | 50 |
|  | **Actual duration (days)** | 27 | 27 | 24 | 27 | 27 | 24 | 27 | 27 | 24 |
| **Patient 8** | **Planned dose (mg)** | 20 | ─ | ─ | 300 | ─ | ─ | 50 | ─ | ─ |
|  | **Actual dose (mg)** | 20 | ─ | ─ | 300 | ─ | ─ | 50 | ─ | ─ |
|  | **Actual duration (days)** | 28 | ─ | ─ | 28 | ─ | ─ | 28 | ─ | ─ |
| **Patient 9** | **Planned dose (mg)** | 10 | ─ | ─ | 300 | ─ | ─ | ─ | ─ | ─ |
|  | **Actual dose (mg)** | 10 | ─ | ─ | 300 | ─ | ─ | ─ | ─ | ─ |
|  | **Actual duration (days)** | 27 | ─ | ─ | 0 | ─ | ─ | ─ | ─ | ─ |
| **Patient 10** | **Planned dose (mg)** | 10 | 10 | 10 | 300 | 300 | 300 | 50 | 50 | ─ |
|  | **Actual dose (mg)** | 5 | 5 | 10 | 200 | 200 | 200 | 50 | 50 | ─ |
|  | **Actual duration (days)** | 28 | 32 | 26 | 28 | 32 | 26 | 28 | 32 | ─ |
| **Patient 11** | **Planned dose (mg)** | 15 | ─ | ─ | 300 | ─ | ─ | 50 | ─ | ─ |
|  | **Actual dose (mg)** | 15 | ─ | ─ | 300 | ─ | ─ | 50 | ─ | ─ |
|  | **Actual duration (days)** | 31 | ─ | ─ | 31 | ─ | ─ | 31 | ─ | ─ |
| **Patient 12** | **Planned dose (mg)** | 20 | 20 | 20 | 300 | 300 | 300 | 50 | 50 | 50 |
|  | **Actual dose (mg)** | 20 | 20 | 15 | 300 | 300 | 300 | 50 | 50 | 50 |
|  | **Actual duration (days)** | 27 | 27 | 26 | 27 | 28 | 26 | 27 | 28 | 26 |

**Abbreviations:** SD =Standard deviation.

**Supplementary Table 1: Criteria for defining dose-limiting toxicity**

| **Toxicity** | **DLT criteria** |
| --- | --- |
| **Hematologic** | |
| Blood and lymphatic system disorders | - Neutropenia CTCAE grade 4*. - Febrile neutropenia CTCAE grade ≥3 (absolute neutrophil count   < 1.0 x 109/L + Fever ≥38.5 ºC).   - Thrombocytopenia CTCAE grade 4 (platelets < 25 x 109/L)*. - Hemorrhagic event CTCAE grade ≥2. - Anemia CTCAE grade 4 for ≥7 days despite packed red blood cell transfusions. |
| **Non-hematologic** | |
| Skin and subcutaneous tissue disorders: Rash and/or photosensitivity | - Rash CTCAE grade ≥ 3 for > 7 consecutive days despite skin toxicity treatment. - Rash CTCAE grade 4. |
| Metabolism and nutrition disorders: Hyperglycemia | - Hyperglycemia grade 3 (fasting plasma glucose > 250 - 400mg/dL) for > 7 consecutive days despite anti-diabetic treatment. - Hyperglycemia grade 4 (fasting plasma glucose > 400mg/dL) confirmed with a repeat fasting plasma glucose within 24 hours. - Hyperglycemia leading to diabetic keto-acidosis, hospitalization for intravenous insulin infusion, or non-ketotic coma. |
| Gastrointestinal disorders | - Diarrhea CTCAE grade ≥ 3 ≥ 48 hours, despite the use of anti- diarrhea therapy. - Nausea/ vomiting CTCAE grade ≥ 3 ≥ 48 hours, despite the use of anti-emetic therapy. |
| Investigations | - Total bilirubin CTCAE grade ≥ 4, coincident direct bilirubin ≥ 0.5 mg/dL. - AST or ALT CTCAE grade ≥ 4 for > 7 consecutive days. - AST or ALT CTCAE grade 4. |
| Cardiac | - QTc prolongation > CTCAE grade 3 confirmed by triplicate ECG 5 minutes apart in the absence of other drugs that are known to cause QT prolongation, that persist following the correction of electrolyte abnormalities, if any. |
| Mood disorders | - CTCAE grade 2 mood alteration that does not resolve to < grade 1 within 14 days despite medical treatment (for anxiety only, if worsened from baseline). - ≥ CTCAE grade 3 mood alterations. |
| Others | - Any CTCAE grade ≥ 3 non-hematologic toxicity for ≥ 7 days. - Any CTCAE grade 4 non-hematologic toxicity. |
| *In two consecutive assessments, the second being considered valid and final.  For toxicities requires a time-window in order to be defined as DLTs (grade 3 non-hematologic toxicity for ≥ 7 consecutive days), a subsequent visit and/or laboratory assessments must be scheduled accordingly; i.e., if a grade 3 non-hematologic toxicity is observed, it must also be observed at least 7 days later, with no intervening lower grades.  **Abbreviations:** ALT = Alanine aminotransferase; AST = Aspartate aminotransferase; CTCAE = Common Terminology Criteria for Adverse Events. | |

**Supplementary Table 5: List of 43 genes implicated in myeloid pathology included in the custom NGS panel.**

| ***GENE*** | **CHR** | **START** | **END** | **COVERAGE (%)** |
| --- | --- | --- | --- | --- |
| *ASXL1* | 20 | 30954122 | 31025231 | 98.59 |
| *BCOR* | X | 39911228 | 39937243 | 100 |
| *BCORL1* | X | 129139130 | 129190192 | 97.03 |
| *CALR* | 19 | 13049460 | 13054786 | 100 |
| *CBL* | 11 | 119077080 | 119170509 | 96.95 |
| *CEBPA* | 19 | 33792147 | 33793455 | 97.57 |
| *CSF3R* | 1 | 36931652 | 36945167 | 100 |
| *DNMT3A* | 2 | 25457047 | 25536929 | 96.81 |
| *EGLN1* | 1 | 231502062 | 231557733 | 89.26 |
| *EPAS1* | 2 | 46525036 | 46611847 | 95.38 |
| *EPOR* | 19 | 11488599 | 11495008 | 93.96 |
| *ETV6* | 12 | 11802967 | 12044078 | 100 |
| *EZH2* | 7 | 148504657 | 148544423 | 100 |
| *FLT3* | 13 | 28578144 | 28644795 | 98.36 |
| *IDH1* | 2 | 209101731 | 209116356 | 100 |
| *IDH2* | 15 | 90627367 | 90635017 | 84.96 |
| *JAK2* | 9 | 5021946 | 5126835 | 100 |
| *KDM6A* | X | 44732709 | 44970753 | 99.01 |
| *KIT* | 4 | 55524176 | 55604767 | 100 |
| *KMT2A* | 11 | 118307241 | 118393002 | 97.7 |
| *KRAS* | 12 | 25362705 | 25398385 | 100 |
| *MPL* | 1 | 43803488 | 43818462 | 99.26 |
| *NF1* | 17 | 29422227 | 29701206 | 99.66 |
| *NPM1* | 5 | 170814868 | 170837656 | 95.27 |
| *NRAS* | 1 | 115251106 | 115258821 | 100 |
| *PHF6* | X | 133511597 | 133559416 | 89.4 |
| *PRPF40B* | 12 | 50017325 | 50038043 | 99.18 |
| *RAD21* | 8 | 117859710 | 117878977 | 100 |
| *RUNX1* | 21 | 36164287 | 36421263 | 98.42 |
| *SETBP1* | 18 | 42281301 | 42643812 | 100 |
| *SF3A1* | 22 | 30730553 | 30752861 | 100 |
| *SF3B1* | 2 | 198256921 | 198299857 | 98.87 |

| *SH2B3* | 12 | 111855923 | 111886159 | 88.57 |
| --- | --- | --- | --- | --- |
| *SMC1A* | X | 53406965 | 53449648 | 98.28 |
| *SRSF2* | 17 | 74732208 | 74733436 | 100 |
| *STAG2* | X | 123156407 | 123234509 | 100 |
| *TET2* | 4 | 106154899 | 106197684 | 100 |
| *THPO* | 3 | 184090090 | 184096202 | 100 |
| *TP53* | 17 | 7572852 | 7579966 | 94.5 |
| *U2AF1* | 21 | 44513191 | 44527685 | 98.86 |
| *VHL* | 3 | 10183360 | 10191667 | 96.58 |
| *WT1* | 11 | 32410545 | 32456973 | 91.46 |
| *ZRSR2* | X | 15808512 | 15841397 | 100 |

**Supplementary Table 4: Adverse events and treatment-related adverse events**

| **Adverse event (n=251)** | **n** | **%** |
| --- | --- | --- |
| Hyperglycemia | 27 | 10.8 |
| Asthenia | 14 | 5.6 |
| Thrombocytopenia | 14 | 5.6 |
| Anemia | 12 | 4.8 |
| ALT increased | 11 | 4.4 |
| Worsening of anemia | 10 | 4.0 |
| Hypocalcemia | 7 | 2.8 |
| AST increased | 4 | 1.6 |
| Hyperbilirubinemia | 4 | 1.6 |
| Insomnia | 4 | 1.6 |
| Slight fever | 4 | 1.6 |
| Worsening of thrombocytopenia | 4 | 1.6 |
| Dyspnea | 3 | 1.2 |
| Edemas | 3 | 1.2 |
| GGT increased | 3 | 1.2 |
| Hyperkalemia | 3 | 1.2 |
| Hyperoxia | 3 | 1.2 |
| Nausea | 3 | 1.2 |
| Upper respiratory infection | 3 | 1.2 |
| **Treatment-related adverse events (n=90)** | **n** | **%** |
| Hyperglycemia | 20 | **22.2** |
| Thrombocytopenia | 12 | **13.3** |
| Anemia | 8 | **8.9** |
| ALT increased | 6 | **6.7** |
| AST increased | 3 | **3.3** |
| GGT increased | 3 | **3.3** |
| Hyperbilirubinemia | 3 | **3.3** |
| Insomnia | 3 | **3.3** |
| Arthralgias | 2 | **2.2** |
| CK increased | 2 | **2.2** |
| Edemas | 2 | **2.2** |
| Meteorism | 2 | **2.2** |
| Peripheral neuropathy in hands | 2 | **2.2** |
| Peripheral neuropathy in legs | 2 | **2.2** |
| Bowen’s disease | 1 | **1.1** |
| Bilateral pleural effusion | 1 | **1.1** |
| Cholestasis | 1 | **1.1** |
| Congestive heart failure | 1 | **1.1** |
| Diarrhea | 1 | **1.1** |
| Dyspepsia | 1 | **1.1** |
| Edema on legs | 1 | **1.1** |
| Facial flushing | 1 | **1.1** |
| Hip fracture | 1 | **1.1** |
| Lipase increased | 1 | **1.1** |
| Lumbar pain | 1 | **1.1** |
| Muscle cramps | 1 | **1.1** |
| Muscular discomfort | 1 | **1.1** |
| Nausea | 1 | **1.1** |
| Pericardial effusion | 1 | **1.1** |
| Pleural effusion | 1 | **1.1** |
| Polyuria | 1 | **1.1** |
| Proximal steroid myopathy | 1 | **1.1** |
| Pulmonary hypertension | 1 | **1.1** |
| Worsening of anemia | 1 | **1.1** |

**Abbreviations:** AE = adverse event; ALT = Alanine aminotransferase; AST = Aspartate aminotransferase; CK = Creatine Kinase; GGT = Gamma-Glutamyltransferase.
